# USP38 Couples Histone Ubiquitination and Methylation via KDM5B to Resolve Inflammation

**DOI:** 10.1002/advs.202002680

**Published:** 2020-10-11

**Authors:** Zhiyao Zhao, Zexiong Su, Puping Liang, Di Liu, Shuai Yang, Yaoxing Wu, Ling Ma, Junyan Feng, Xiya Zhang, Chenglei Wu, Junjiu Huang, Jun Cui

**Affiliations:** ^1^ MOE Key Laboratory of Gene Function and Regulation State Key Laboratory of Biocontrol School of Life Sciences Sun Yat‐sen University Guangzhou Guangdong 510006 China; ^2^ Department of Internal Medicine Guangzhou Institute of Pediatrics Guangzhou Women and Children's Medical Center Guangzhou Guangdong 510623 China

**Keywords:** deubiquitinase, histone modification, inflammation, KDM5B, ubiquitination, USP38

## Abstract

Chromatin modifications, such as histone acetylation, ubiquitination, and methylation, play fundamental roles in maintaining chromatin architecture and regulating gene transcription. Although their crosstalk in chromatin remodeling has been gradually uncovered, the functional relationship between histone ubiquitination and methylation in regulating immunity and inflammation remains unclear. Here, it is reported that USP38 is a novel histone deubiquitinase that works together with the histone H3K4 modifier KDM5B to orchestrate inflammatory responses. USP38 specifically removes the monoubiquitin on H2B at lysine 120, which functions as a prerequisite for the subsequent recruitment of demethylase KDM5B to the promoters of proinflammatory cytokines *Il6* and *Il23a* during LPS stimulation. KDM5B in turn inhibits the binding of NF‐*κ*B transcription factors to the *Il6* and *Il23a* promoters by reducing H3K4 trimethylation. Furthermore, USP38 can bind to KDM5B and prevent it from proteasomal degradation, which further enhances the function of KDM5B in the regulation of inflammation‐related genes. Loss of *Usp38* in mice markedly enhances susceptibility to endotoxin shock and acute colitis, and these mice display a more severe inflammatory phenotype compared to wild‐type mice. The studies identify USP38‐KDM5B as a distinct chromatin modification complex that restrains inflammatory responses through manipulating the crosstalk of histone ubiquitination and methylation.

## Graphical abstract

USP38 is a novel histone deubiquitinase of H2B that couples the regulation of histone ubiquitination (H2Bub) and methylation (H3K4me3) with KDM5B to selectively inhibit the transcription of the proinflammatory cytokines and prevent excessive inflammation. USP38 deficiency in mice enhances the inflammatory response, and renders animals more susceptible to acute inflammation and dextran sulfate sodium‐induced acute colitis.

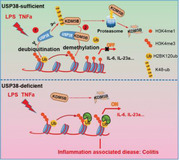

## 1 Introduction

Inflammation is a conserved defense mechanism used by hosts to fight against injury or disease.^[^ ^1^ ^]^ Several types of innate immune cells, including macrophages and dendritic cells (DCs), play important roles in regulating the inflammatory response through pattern recognition receptor (PRR)‐mediated signaling.^[^ ^2^ ^]^ Upon recognition of pathogen‐associated molecular patterns (PAMPs), PRRs trigger the intracellular signaling pathways that induce potent host innate immune responses and prompt the maturation of immune cells. DCs and macrophages produce various cytokines that regulate the immune response and promote inflammatory responses.^[^ ^3^, ^4^ ^]^ Although inflammation is important for the initiation of protective immunity, dysregulated inflammation can lead to tissue destruction, autoimmune disease, and tumor progression owing to the excessive production of proinflammatory cytokines.^[^ ^5^, ^6^, ^7^, ^8^ ^]^ Therefore, the inflammatory response must be tightly regulated.

Inflammation could be induced and controlled by the regulation of PRR‐mediated signaling networks, which involves the transcription factor nuclear factor‐*κ*B (NF‐*κ*B) signaling and inflammasome pathway. PRRs, such as Toll‐like receptors (TLRs), can initiate the activation of NF‐*κ*B signaling to induce the expression of a variety of proinflammatory cytokines, such as IL‐6, TNF‐*α*, and IL‐23*α*.^[^ ^9^ ^]^ Some proinflammatory cytokines, including IL‐1*β* and IL‐18, require further processing by inflammasomes to become their mature and secreted forms.^[^ ^10^ ^]^ Numerous studies have shown the strict and hierarchical regulation of the NF‐*κ*B signaling cascade at multiple levels.^[^ ^11^ ^]^ Accumulating evidence has also shown that TLR‐mediated NF‐*κ*B signaling and inflammatory responses could be regulated at the chromatin level.^[^ ^12^, ^13^ ^]^ It has been reported that the expression of IL‐6 could be regulated by histone deacetylation.^[^ ^14^ ^]^ Thus, chromatin remodeling by histone modifications might play an important role in regulating NF‐*κ*B signaling as well as inflammation.

Various studies have demonstrated the important role of histone acetylation, methylation, and ubiquitination in transcriptional initiation and elongation.^[^ ^15^, ^16^, ^17^ ^]^ Histone acetylation and methylation have been illustrated to be critical for activating or repressing proinflammatory cytokine transcription in inflammation.^[^ ^12^ ^]^ Histone acetyltransferase p300 mediates the acetylation of H3 at lysine 18 and lysine 27 (H3K18/27ac) to activate the expression of proinflammatory genes,^[^ ^18^ ^]^ whereas deacetylase HDAC1/2 inhibits *Il6* transcription to restrain the inflammatory response.^[^ ^19^ ^]^ It has also been reported that histone demethylase Jmjd2d removed H3K9me2/3 histone marks on the *Il12*/*Il23* promoter for LPS‐induced Th1 and Th7 differentiation.^[^ ^20^, ^21^ ^]^ However, whether the ubiquitination of histone functions in the context of the inflammatory response is currently unknown.

Accumulating evidence indicates the central role of monoubiquitination of H2B at lysine 120 (H2Bub) in the regulation of chromatin dynamics since several studies showed that H2Bub is a prerequisite for the methylation of H3K4 and H3K79,^[^ ^22^, ^23^, ^24^, ^25^, ^26^ ^]^ which are the active signals for gene transcription.^[^ ^27^ ^]^ H2Bub is reported to recruit the histone methyltransferase complex DOT1L and COMPASS, which are involved in the methylation of H3K79 and H3K4, respectively.^[^ ^24^, ^28^, ^29^, ^30^, ^31^, ^32^, ^33^ ^]^ However, the detailed cross‐regulatory mechanisms of H2Bub and H3K4me3 still need to be resolved.

Here, we identified a novel histone H2B deubiquitinase, USP38, as a crucial regulator that mediates the inflammatory response by controlling the selective expression of proinflammatory cytokines. Knockout (KO) of *Usp38* in mice causes excessive inflammatory responses which enhance the symptoms of acute colitis and lung injury in response to endotoxin shock. USP38 specifically removes H2Bub at the promoters of *Il6* and *Il23a *during LPS stimulation and facilitates the recruitment of demethylase KDM5B (also as Jarid1B), a specific demethylase on di‐ or trimethylation of H3K4,^[^ ^34^, ^35^ ^]^ for subsequent H3K4 demethylation at the same promoters. In addition, our results show that USP38 could also stabilize KDM5B through removing its K48‐linked ubiquitin chains, which further enhances the KDM5B‐mediated inhibition of *Il6* and *Il23a* expression. Our data highlighted a new epigenetic regulatory mechanism in which USP38‐KDM5B couples histone ubiquitination and methylation to resolve inflammation via selective regulation of proinflammatory cytokines.

## 2 Results

### 2.1 *Usp38* Deficiency Promotes Inflammatory Responses In Vitro and In Vivo

**Figure 1:**
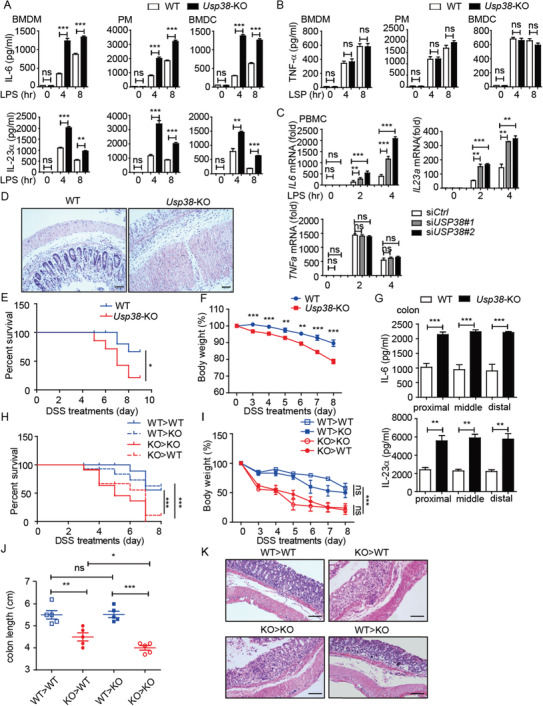
Loss of USP38 enhances inflammatory responses in vitro and in vivo. A) Protein levels of IL‐6 and IL‐23*α* in BMDMs, BMDCs, and PMs from wild‐type (WT) and *Usp38*‐knockout (KO) mice during LPS stimulation for the indicated time points. B) Protein level of TNF‐*α* in BMDMs, BMDCs, and PMs from WT and *Usp38*‐KO mice during LPS stimulation for the indicated time points. C) Expression level of *IL6*, *IL23a*, and *TNFa* mRNA in PBMCs transfected with *USP38*‐specific siRNAs (si*USP38#1*, si*USP38#2*) or control (si*Ctrl*) siRNA under LPS treatment for the indicated time points. D–F) WT and *Usp38*‐KO mice were challenged with 2.5% dextran sulfate sodium (DSS) for 7 d and sacrificed at day 8. KO, *n* = 6; WT, *n* = 6. Histopathology of the distal colon (D), survival (E), and weight changes (F) in WT and *Usp38*‐KO mice. G) Protein levels of IL‐6 and IL‐23*α* in colon tissue from (D). H–K) Survival, weight changes, colon length, and histopathology of the distal colon in the indicated bone marrow chimera mice (*n* = 8–12/group). Data in (A)–(C) are presented as the means ± SEM of at least three biological experiments. Data in (E)–(G) are presented as the means ± SD of the experiment with six mice per group. Data in (H)–(J) are presented as the means ± SD of the experiment with 8–12 mice per group. Data in (D)–(K) are representative of three independent experiments. *p < 0.05, **p < 0.01, ***p < 0.001, ns, no significant difference, versus the wild type or control group with the same treatment (Student's *t*‐test in (A)–(C), (G), (F), (I), (J) and Mantel–Cox test and Gehan–Breslow–Wilcoxon test in (E) and (H)).

We found that bone marrow‐derived DCs (BMDCs), peritoneal macrophages (PM), and bone marrow‐derived macrophages (BMDMs) with a deficiency of *Usp38* (*Usp38*‐KO) all maintained higher mRNA levels of *Il6* and *Il23a* as well as higher IL‐6 and IL‐23*α* secretion at the early phase of lipopolysaccharide (LPS, a TLR4 ligand) stimulation than the wild‐type (WT) cells (**Figure** **1**A and Figure S1A, Supporting Information). In contrast, neither *Tnfα* mRNA nor its secretion level showed a difference with LPS treatment in all tested cells (Figure 1B and Figure S1B, Supporting Information). We further confirmed the inhibitory role of USP38 on *Il6* and *Il23a* but not *Tnfα* expression in human peripheral blood mononuclear cells (PBMCs) (Figure 1C and Figure S1C, Supporting Information).

To assess the biological significance of USP38 in inflammatory responses in vivo, we used an endotoxin shock model. With peritoneal injection of LPS, *Usp38*‐KO mice produced much more IL‐6 and IL‐23*α*, and their lungs exhibited more severe tissue damage and diffuse inflammation (Figure S1D,E, Supporting Information). These results indicate that USP38 might function as a negative regulator of inflammation. To further confirm the role of USP38 in the control of inflammation, we used a dextran sulfate sodium (DSS)‐induced acute colitis mouse model (Figure S1F, Supporting Information), which is mainly dependent on the dysregulation of the innate immune system.^[^ ^36^ ^]^ We found that *Usp38*‐KO mice were more susceptible to DSS‐induced colitis by showing exacerbated colon inflammation, along with higher mortality, exaggerated weight loss, shorter colon length, higher production of IL‐6 and IL‐23*α* in colon tissue and blood, more severe disruption of mucosal structures in histological analysis of the colons, and higher clinical score in *Usp38*‐KO mice compared to WT mice (Figure 1D–G and Figure S1F–J, Supporting Information). Overall, these results suggest an inhibitory function of USP38 in controlling inflammatory responses in vitro and in vivo.

### 2.2 The Pathogenesis in *Usp38*‐KO Mice Is Derived from Hematopoietic Sources

It has been reported that the development of colitis might be mainly due to the proinflammatory cytokines secreted by enterocytes and immune cells.^[^ ^37^ ^]^ Various types of T cells have been reported to play crucial roles in intestinal inflammation.^[^ ^38^, ^39^, ^40^ ^]^ We first checked the developmental defects of innate and adaptive immune cells, and no significant differences were observed in *Usp38*‐KO mice, except that CD4^+^ T cells were slightly decreased along with a minor increase in CD8^+^ T cells (Figure S2, Supporting Information), which was consistent with a previous report.^[^ ^36^ ^]^ To investigate the relevant cell compartment responsible for colon inflammation in *Usp38*‐KO mice, we next generated *Usp38*‐KO and WT chimeric mice with adoptive bone marrow transplantation (Figure S1K, Supporting Information). Among bone marrow recipients that were treated with DSS, WT mice and *Usp38*‐KO mice receiving *Usp38*‐KO bone marrow (referred to as KO>WT and KO>KO) showed a similar trend, as did the WT mice and *Usp38*‐KO mice receiving WT bone marrow (referred to as WT>WT and WT>KO). Both the KO>WT and KO>KO groups presented with significantly more severe symptoms of colitis compared to the WT>WT and WT>KO groups. Differences in the mouse survival ratio (Figure 1H), weight loss (Figure 1I), and colon length (Figure 1J and Figure S1L, Supporting Information) all reached statistical significance by day 8 after DSS administration. The marked improvement in the clinical manifestation of colitis was further confirmed by hematoxylin and eosin (H&E)‐stained sections of intestinal mucosa. There were fewer signs of severe histopathology of the lamina propria in the WT>WT and WT>KO groups, while the KO>WT and KO>KO groups presented with extensive crypt destruction and edema due to the loss of USP38 in the transplanted bone marrow (Figure 1K). Interestingly, we found that although the difference of colon length between KO>KO and KO>WT is smaller than that between KO>WT and WT>WT, the colon of KO>KO still had shorter length than KO>WT, indicating that despite its major role in the bone marrow‐derived cells, USP38 might also function in other structural cell types, which contribute to the colitis progressing. To further confirm whether these USP38‐regulated cytokines are responsible for the DSS‐induced colitis in USP38‐KO mice, we neutralized IL‐6 and IL‐23 by peritoneal injection of anti‐IL‐6/IL‐23 antibodies (Abs) in WT or USP38‐KO colitis mice. Consistently, blockage of IL‐6/IL‐23 can protect the mice from colon damage and colitis‐induced death (Figure S1M,N, Supporting Information). Collectively, these results highlight a crucial role for USP38‐regulated cytokines in protection against the development of inflammation‐induced colitis in bone marrow‐derived cells.

### 2.3 Identification of USP38‐Regulated Pathways in Inflammation

**Figure 2:**
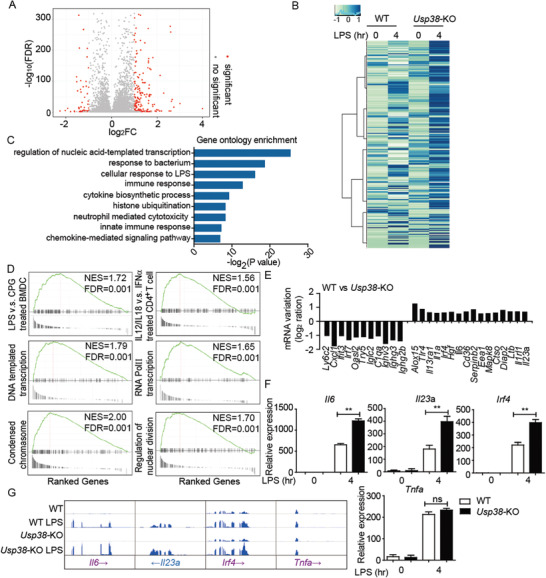
Effect of USP38 on specific gene expression during the inflammatory response. A) USP38 regulates inflammation‐relevant target genes, presented as a volcano plot of genes with differential expression after LPS stimulation in wild‐type (WT) and *Usp38*‐knockout (KO) BMDMs. B) Heat map view of top and bottom gene list of RNA‐sequence data sets. Microarray analysis for total RNA was performed for WT and *Usp38*‐KO BMDMs with or without LPS treatment. C) Gene ontology enrichment analysis of the USP38‐dependent genes in B (−log_2_ *P* values). D) GSEA of differentially expressed genes in *Usp38*‐KO BMDMs with LPS treatment and enrichment of different signatures. FDR (*q*‐value) was shown. E) The log2 ratio of mRNA variations in *Usp38*‐KO BMDMs after LPS stimulation for 4 h. F) RT‐qPCR analysis of USP38‐dependent genes in WT and *Usp38*‐KO BMDMs with or without LPS treatment. G) RNA‐seq analysis of representative inflammatory response genes in WT and *Usp38*‐KO BMDMs with or without LPS treatment. Data in (F) are presented as the means ± SEM of at least three biological experiments. *p < 0.05, **p < 0.01, ***p < 0.001, ns, no significant difference, versus the wild type or control group with the same treatment (Student's *t*‐test).

We then used global RNA‐sequencing analysis to identify cells and pathways regulated by USP38. Analysis of differentially expressed genes (with a cutoff of 1.5‐fold; adjusted *P* value < 0.05) identified 245 upregulated genes and 403 downregulated genes in response to *Usp38* deficiency in BMDMs during LPS stimulation (**Figure** **2**A,B). Gene‐ontology annotation of these genes showed significant enrichment for immune‐related functions (log_2_ *P* < 2^−8^ to log_2_ *P* < 2^−25^), including “response to bacterium” (Figure 2C).

Gene‐set‐enrichment analyses (GSEA) using >1900 cell‐specific and pathway‐specific immune signatures identified enrichment (FDR < 0.01) for signatures associated with responses of various cells to LPS, responses of CD4^+^ T cells to cytokine stimulation, and responses to IFN‐*α* (Figure 2D). Surprisingly, some signatures associated with DNA‐templated transcription and RNA Pol II transcription as well as the regulation of chromosome and nuclear division have also been detected in GSEA enrichment (Figure 2D). These data indicate that USP38 might be involved in the cross‐regulation of immunity and chromatin remodeling.

To investigate the role of USP38 in regulating inflammatory responses, we identified 11 downregulated genes and 16 upregulated genes in *Usp38*‐KO BMDMs (Figure 2E) from RNA sequencing (RNA‐seq) data. Among the upregulated genes, we found that USP38 specifically inhibits a particular set of proinflammatory cytokines. Additional RNA‐expression studies (real‐time quantitative PCR (RT‐qPCR)) of these upregulated genes (*Il6*, *IL23a*, and *Irf4*) were confirmed in WT and *Usp38*‐KO BMDMs (Figure 2F). We further identified the principal USP38‐regulated genes and associated signatures corresponding to inflammatory responses (*Il6*, *Il23a*, *Ifit3*, and *Irf4*) from WT and *Usp38*‐KO BMDMs with or without LPS treatment datasets (Figure 2G). In addition, we found the expression of *Tnfa* showed no difference between WT and *Usp38*‐KO groups under LPS stimulation. These results indicate that USP38 negatively regulates the inflammatory response during LPS stimulation by specifically controlling the expression of inflammatory genes such as *Il6* and *Il23a*.

### 2.4 USP38 Negatively Regulates the Recruitment of NF‐*κ*B Transcription Factors to the *Il6* and *Il23a* Promoters

To elucidate the molecular mechanism by which USP38 negatively regulates the induction of genes encoding inflammatory cytokines, we investigated the role of USP38 in TLR‐mediated activation of NF‐*κ*B and mitogen‐activated protein kinase (MAPK) signaling. *Usp38*‐KO BMDMs and BMDCs did not show an appreciable difference in the LPS‐stimulated phosphorylation of IKK or its downstream substrates or MAPKs (p38, ERK, and JNK) compared with that in WT cells (Figure S3A,B, Supporting Information). Consistently, no significant difference was observed in Flag‐USP38‐inducible THP‐1 cells stimulated with LPS and either with or without doxycycline (DOX) (Figure S3C, Supporting Information). In addition, we found that the phosphorylation of p65 showed no difference in WT and *USP38*‐silenced PBMCs (Figure S3D, Supporting Information). However, we were surprised to observe that the binding of USP38 to chromatin was increased with LPS stimulation (Figure S3E, Supporting Information), indicating that USP38 might affect inflammation through epigenetic regulation.

**Figure 3:**
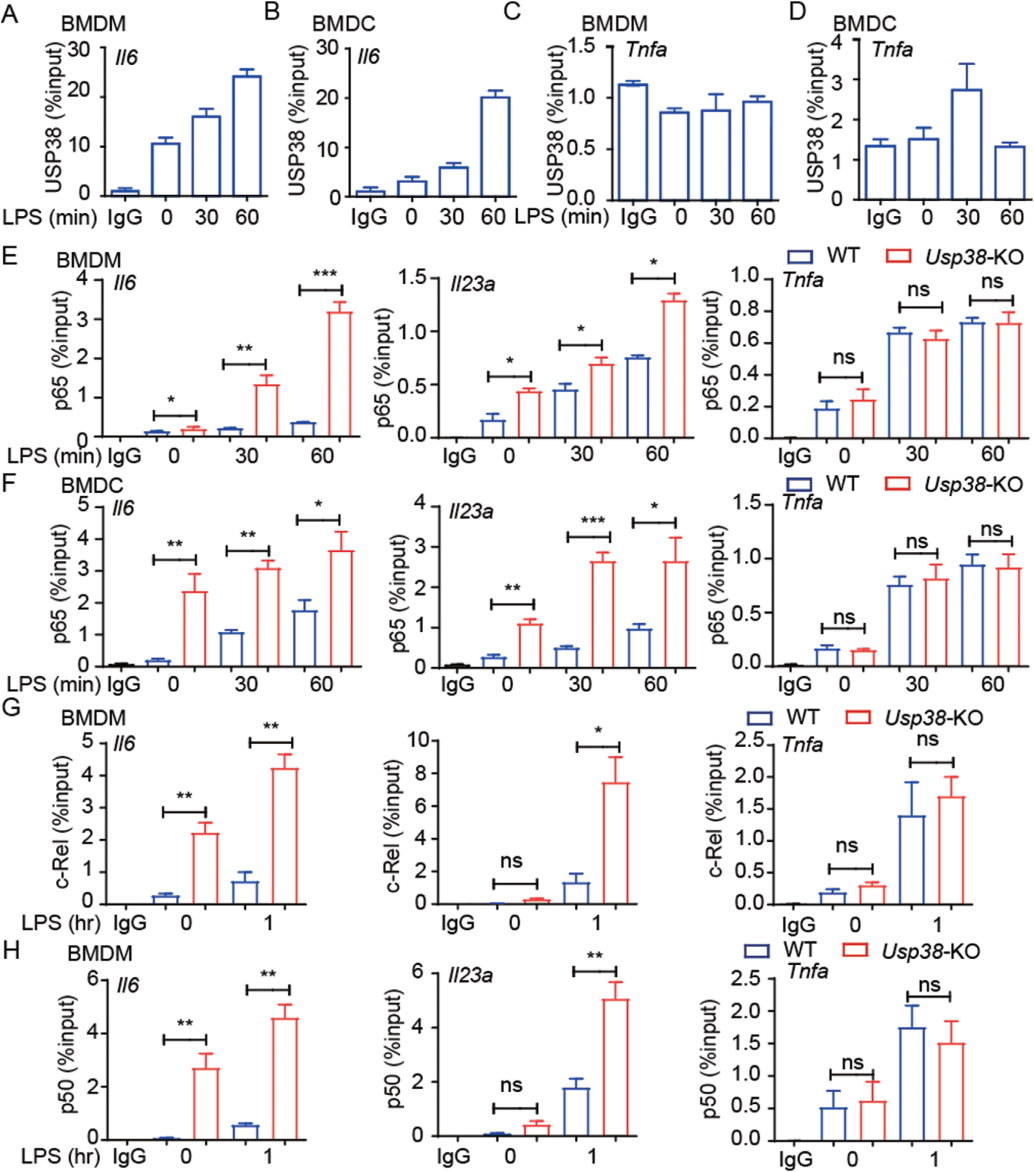
USP38 negatively regulates the recruitment of NF‐*κ*B transcription factors to the *Il6* and *Il23a* promoters. ChIP followed by quantitative PCR (ChIP‐qPCR) of USP38 at the A,B) *Il6* and C,D) *Tnfa* promoters in A,C) BMDMs and B,D) BMDCs during LPS stimulation for the indicated time points. IP, immunoprecipitation. E,F) ChIP‐qPCR of p65 at the *Il6*, *Tnfa*, and *Il23a* promoters in wild‐type (WT) and *Usp38*‐knockout (KO) BMDMs and BMDCs during LPS stimulation for the indicated time points. G,H) ChIP‐qPCR of c‐Rel and p50 at the *Il6*, *Tnfa*, and *Il23a* promoters in WT and *Usp38*‐KO BMDMs with or without LPS treatment; results are presented relative to those of 1% input DNA. Data in (A)–(H) are presented as the means ± SEM of three independent biological experiments. *p < 0.05, **p < 0.01, ***p < 0.001, ns, no significant difference, versus the WT or control group with the same treatment (Student's *t*‐test).

Since USP38 specifically affects the expression of IL‐6 but not TNF‐*α*, we then investigated whether USP38 could directly target the *Il6* gene for epigenetic regulation. Using chromatin immunoprecipitation (ChIP), we found an increase in the binding signal of USP38 to the *Il6* promoter with LPS stimulation in both BMDMs and BMDCs (**Figure** **3**A,B). In contrast, the recruitment of USP38 to the *Tnfα* promoter was barely detectable in these cells (Figure 3C,D). Because p65 has an important role in the transcription of the genes encoding IL‐6, TNF‐*α*, and IL‐23*α*, we then investigated whether USP38 regulates the TLR‐stimulated recruitment of p65 to the *Il6, Tnfa*, and *Il23a* promoters. The binding of p65 to the promoters of *Il6* and *Il23a* was increased by LPS stimulation in *Usp38*‐KO BMDMs compared to those in WT BMDMs or BMDCs, while the recruitment of p65 to the *Tnfa* promoter was barely changed (Figure 3E,F).

We next assessed the recruitment of other NF‐*κ*B factors to promoters of cytokine‐encoding genes. In *Usp38*‐KO BMDMs, the binding of c‐Rel and p50 to the promoters of *Il6* and *Il23a*, but not *Tnfa*, was significantly increased (Figure 3G,H). Thus, our results demonstrated that USP38 specifically inhibited the recruitment of NF‐*κ*B factors (p65, c‐Rel, p50) to the *Il6* and *Il23a* promoters after LPS stimulation.

### 2.5 Regulation of Histone Ubiquitination and Methylation by USP38

**Figure 4:**
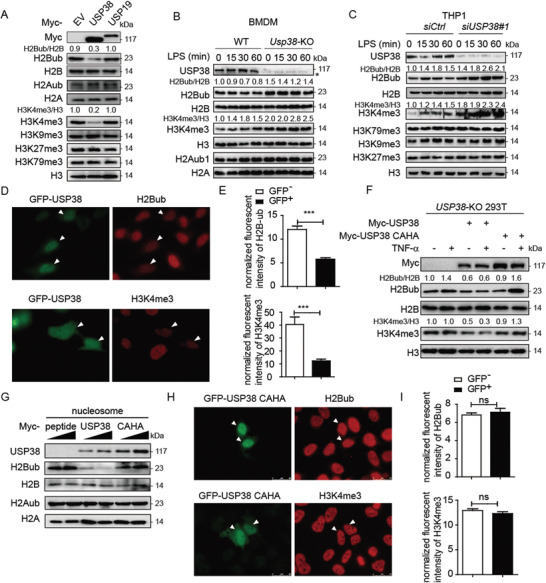
USP38 specifically reduces monoubiquitination of H2B and trimethylation of H3K4. A) Immunoblot analysis of extracts of HEK293T cells transfected with Myc‐empty vector (EV), Myc‐tagged USP38, or Myc‐USP19 with the indicated antibodies. The protein levels of H2Bub/H2B and H3K4me3/H3 were quantified by ImageJ software (NIH). B,C) Immunoblot analysis of H2Bub/H2B and H3K4me3/H3 in wild‐type (WT) and *Usp38*‐knockout (KO) BMDMs or control and *USP38*‐silenced THP1 cells with LPS treatment for the indicated time points. D,E) Immunofluorescence analysis of H2BK120ub (H2Bub) and H3K4me3 with GFP‐USP38 in HeLa cells. The relative intensity of fluorescence signals of H2Bub and H3K4me3 in GFP‐USP38 transfected cells (GFP^+^) versus control cells (GFP^−^) in the same image frame was analyzed by using ImageJ software (NIH). F) Immunoblot analysis of H2Bub/H2B and H3K4me3/H3 in *USP38*‐KO 293T cells with Myc‐vector, Myc‐USP38, or Myc‐USP38 CAHA overexpression under TNF*α* treatment. G) In vitro deubiquitylation assay of H2B by incubating purified nucleosomes with increasing amounts of purified Myc‐USP38, Myc‐USP38 CAHA mutant, or Myc‐peptide. H,I) Immunofluorescence analysis of H2Bub and H3K4me3 with the GFP‐USP38‐CAHA mutant in HeLa cells. The experimental setting is similar to that in (D) and (E). Data in (A)–(D) and (F)–(H) are representative of three independent biological experiments. Data in (E) and (I) are presented as the means ± SEM of at least three independent experiments with 50 cells/experiment. ***p < 0.001, ns, no significant difference, versus the control cells (Student's *t*‐test).

Accumulating evidence has shown that histone modifications, such as ubiquitination and methylation, are associated with gene transcription and silencing.^[^ ^23^, ^41^, ^42^, ^43^ ^]^ We next investigated whether USP38 directly affects histone modifications. By overexpressing USP38 (USP19 serving as a control due to its cytosol localization), we found that USP38 could specifically reduce the monoubiquitination of H2B at lysine 120 (K120) (H2Bub) and trimethylation of H3 at lysine 4 (H3K4me3) (**Figure** **4**A). Consistent with these results, USP38 deficiency in THP1 cells or BMDMs showed markedly increased H2Bub and H3K4me3 (Figure 4B,C), which can remodel the chromatin for transcription factor binding and subsequent gene transcription.^[^ ^44^ ^]^ We next transfected HeLa cells with GFP‐fused USP38 and used immunofluorescence analysis to detect H2Bub and H3K4me3 levels. The cells with USP38 overexpression had reduced levels of both H2Bub and H3K4me3 compared to adjacent cells without ectopic USP38 (with GFP‐vector overexpression in HeLa cells as a control) (Figure 4D,E and Figure S4A, Supporting Information). Knockdown of *USP38* by *USP38*‐specific shRNA consistently increased H2Bub and H3K4me3 levels (Figure S4B, Supporting Information). Since USP38 is a deubiquitinase, we next investigated whether it affects histone modifications through its protease activity. In *USP38*‐KO 293T cells, TNF*α* treatment induced the level of both H2Bub and H3K4me3,^[^ ^45^ ^]^ while these elevated histone modifications were eliminated with USP38 overexpression (Figure 4F). However, the USP38 inactive mutant (CAHA) failed to reduce H2Bub and H3K4me3 level any further (Figure 4F). We further purified recombinant USP38 and its inactive mutant and incubated them with purified nucleosomes to examine its deubiquitination activity toward H2B mono‐ubiquitination at K120 in vitro; we found that USP38 can specifically cleave the monoubiquitin on H2B but has no effect on the H2A modification (Figure 4G). Further immunofluorescence analysis showed that the USP38 CAHA mutant lost its ability to affect H2Bub and H3K4me3 levels (Figure 4H,I). Therefore, USP38 functions as a deubiquitinase for H2Bub, which reduces the monoubiquitination of H2B and trimethylation of H3K4.

### 2.6 USP38 Selectively Alters Histone Modifications at the *Il6 and Il23a* Promoters

**Figure 5:**
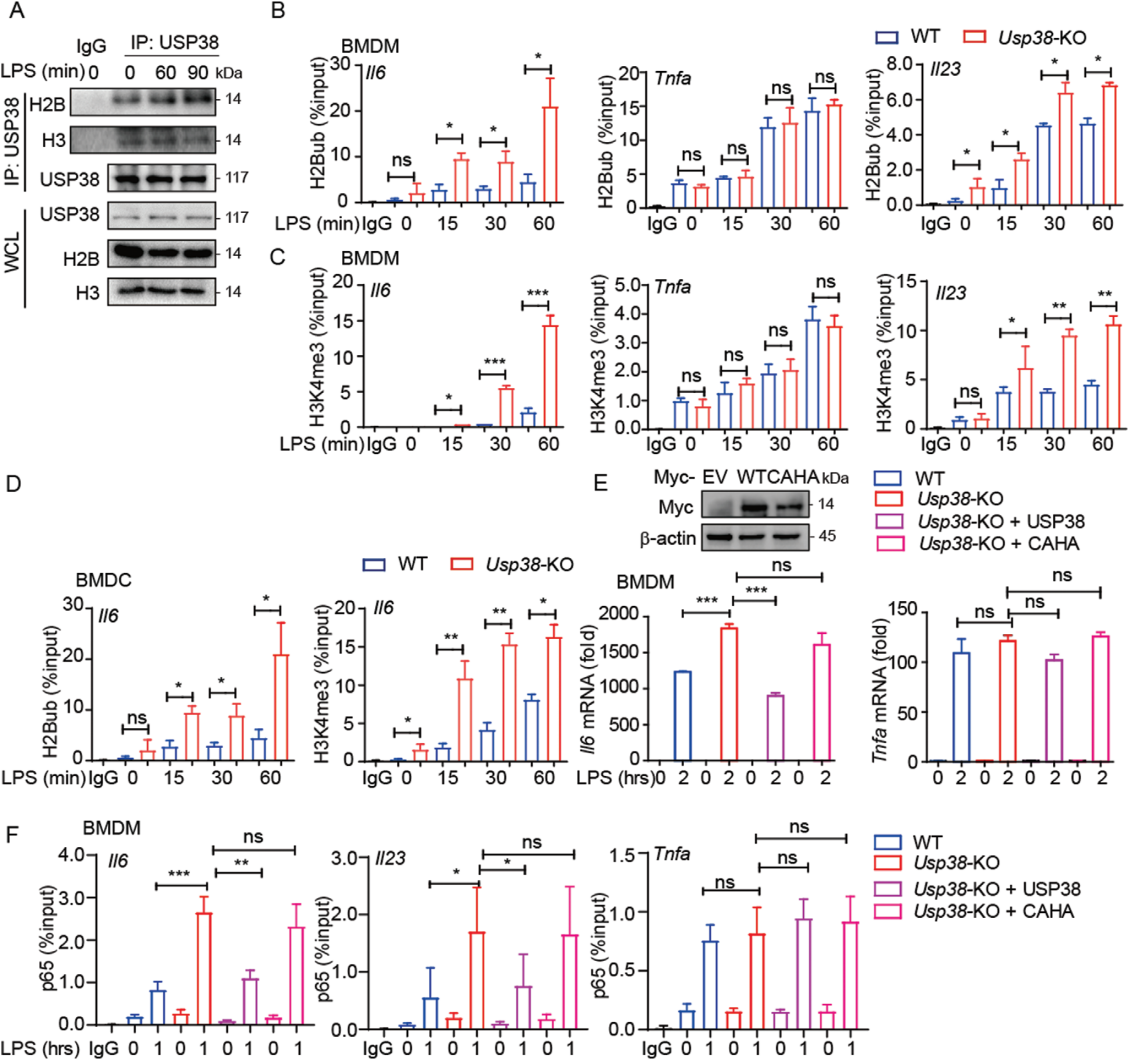
USP38 functions as a deubiquitinase to facilitate histone demethylation at the *Il6* and *Il23a* promoters. A) Coimmunoprecipitation and immunoblot analysis of extracts of BMDMs with LPS treatment for the indicated time points. WCL, whole‐cell lysates. B,C) ChIP‐qPCR of H2Bub and H3K4me3 at the *Il6*, *Tnfa*, and *Il23a* promoters in wild‐type (WT) and *Usp38*‐knockout (KO) BMDMs during LPS stimulation for the indicated time points. D) ChIP‐qPCR of H2Bub and H3K4me3 at the *Il6* promoter in WT and *Usp38*‐KO BMDCs during LPS stimulation for the indicated time points. E) Immunoblot analysis and RT‐PCR analysis of the response to reintroducing Myc‐empty vector (EV), Myc‐USP38, or Myc‐USP38‐CAHA mutant to *Usp38*‐KO BMDMs under LPS treatment. F) ChIP‐qPCR of p65 at the *Il6*, *Il23a*, and *Tnfa* promoters of WT or *Usp38*‐KO BMDMs reintroduced with Myc‐EV, Myc‐USP38, or Myc‐USP38‐CAHA mutant under LPS treatment; results are presented relative to those of 1% input DNA. Data in (A) are representative of three independent biological experiments. Data in (B)–(F) are presented as the means ± SEM of at least three biological experiments. *p < 0.05, **p < 0.01, ***p < 0.001, ns, no significant difference, versus the WT or control group with the same treatment (Student's *t*‐test).

Since a previous study^[^ ^46^ ^]^ showed that USP38 can specifically bind to chromatin, we then detected the interaction between USP38 and H2B or H3. With LPS stimulation, we observed that the interaction between USP38 and H2B, but not USP38 and H3, gradually increased (**Figure** **5**A). We next assessed whether USP38 affects relative histone modifications at the promoters of *Il6*, *Tnfa*, and *Il23a*. By using a ChIP‐qPCR assay, we found that H2Bub and H3K4me3 levels at the *Il6* and *Il23a* promoters were markedly higher with LPS stimulation in *Usp38*‐KO BMDMs than in WT cells, while no detectable difference was found at the *Tnfa* promoter (Figure 5B,C). Similar observations were found in BMDCs (Figure 5D). Furthermore, we reintroduced WT USP38 or the USP38 CAHA mutant into *Usp38*‐KO BMDMs and found that the higher expression of *Il6* in *Usp38*‐KO cells could be reversed by the expression of WT USP38 but not by its inactive mutant (Figure 5E). In addition, ChIP‐qPCR showed that the recruitment of p65 to the *Il6* and *Il23a* promoters was inhibited in *Usp38*‐KO BMDMs by reintroducing WT USP38 but not by introducing its CAHA mutant (Figure 5F), indicating that USP38 affects gene transcription via its deubiquitinase activity. Therefore, USP38 functions as a deubiquitinase to specifically regulate histone modifications of H2B and H3 at the promoters of *Il6 and Il23a*.

### 2.7 USP38 Recruits KDM5B to Repress *Il6* and *Il23a* Expression through Histone Demethylation

**Figure 6:**
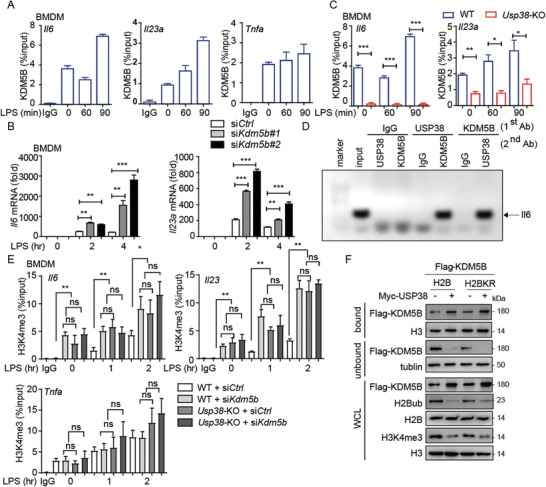
USP38 recruits KDM5B to specifically repress the transcription of *Il6* and *Il‐23a*. A) ChIP‐qPCR of KDM5B at the *Il6*, *Tnfa*, and *Il23a* promoters in BMDMs under LPS treatment for the indicated time points. B) The level of *Il6* and *Il23a* mRNA in *Kdm5b*‐silenced BMDMs using *Kdm5b*‐specific siRNAs (si*Kdm5b#1*, si*Kdm5b#2*) under LPS treatment for the indicated time points. C) ChIP‐qPCR of KDM5B at the *Il6* and *Il23a* promoters in wild‐type (WT) or *Usp38*‐knockout (KO) BMDMs under LPS treatment for the indicated time points. D) Re‐ChIP‐PCR assay of the USP38‐KDM5B interaction at *Il6* promoter in BMDMs after LPS stimulation for 2 h. First‐round ChIP (first antibody, Ab) against USP38, KDM5B, or IgG. Eluted samples were re‐ChIPed with the indicated second antibodies. Lane 2 was input. E) ChIP‐qPCR of H3K4me3 at the *Il6* and *Il23a* promoters in WT or *Usp38*‐KO BMDMs with *Kdm5b*‐specific siRNA or control siRNA under LPS treatment for the indicated time points; results are presented relative to those of 1% input DNA. F) Immunoblot analysis of KDM5B, H2Bub/H2B, and H3K4me3/H3 in chromatin bound and unbound components of 293T cells with overexpression of Myc‐empty vector (EV), Myc‐USP38, or Myc‐USP38 CAHA mutant along with Flag‐H2B or Flag‐H2BK120R mutant. Data in (A)–(C) and (E) are presented as the means ± SEM of at least three biological experiments. Data in (D) and (F) are representative of three independent biological experiments. *p < 0.05, **p < 0.01, ***p < 0.001, ns, no significant difference, versus the WT or control group with the same treatment (Student's *t*‐test).

Monoubiquitination of H2B is reported to promote H3 methylation at K4 and K79 by recruiting the histone methyltransferase complex COMPASS or DOT1L, respectively.^[^ ^47^, ^48^ ^]^ However, the detailed mechanism of the regulation of H3 methylation through H2B deubiquitination is still unclear. Current studies show that KDM5B is an important demethylase that specifically removes the histone marks on H3K4me2 and H3K4me3, while these histone marks are required for transcriptional activation.^[^ ^34^ ^]^ Thus, we assumed that H2Bub might serve as an inhibitory signal for KDM5B to mediate the removal of H3K4me3, and USP38 could reverse this process by cleaving H2Bub. With a ChIP‐qPCR assay, we first found that LPS treatment specifically induced the binding of KDM5B to the *Il6* and *Il23a* promoters but not the *Tnfa* promoter in BMDMs, which is similar to the pattern of USP38 (**Figure** **6**A). In addition, we found that silencing *Kdm5b* significantly enhanced the mRNA levels of *Il6* and *Il23a* but not *Tnfa* during LPS stimulation (Figure 6B and Figure S5A,B, Supporting Information), suggesting the involvement of KDM5B in the gene‐specific regulation of inflammation responses. We next confirmed that the binding of KDM5B to the promoters of *Il6* and *Il23a* was greatly impaired in *Usp38*‐KO BMDMs (Figure 6C), indicating that the recruitment of KDM5B to the *Il6* and *Il23a* promoters was dependent on USP38. We further performed sequential ChIP (re‐ChIP) and showed that USP38 and KDM5B formed a complex on the *Il6* promoter in response to LPS treatment in BMDMs (Figure 6D). We next showed that deficiency of either *Kdm5b* or *Usp38* specifically enhanced the recruitment of H3K4me3 to *Il6* and *Il23a* promoters (Figure 6E) and subsequently increased the mRNA levels of *Il6* and *Il23a* by LPS treatment (Figure S5C, Supporting Information). Together, these data suggest that both USP38 and KDM5B were required for the inhibition of H3K4me3 at the promoters of *Il6* and *Il23a* to retrain inflammatory responses.

We next investigated whether H2Bub affects the recruitment of KDM5B recruitment. We introduced Flag‐tag H2BK120R mutant into HEK293T cells to specifically reduce the H2Bub level, as previously reported,^[^ ^49^ ^]^ and observed increased binding of KDM5B to chromatin, indicating that the binding of KDM5B might be related to the level of H2B monoubiquitination, and USP38 might enhance this process by reducing H2Bub levels (Figure 6F). Interestingly, we found that USP38 overexpression could still enhance the recruitment of KDM5B in the presence of H2BK120R (H2BKR), and the KDM5B protein level was increased by USP38 overexpression (Figure 6F). These results suggest that USP38 might have additional functions to affect KDM5B recruitment to chromatin.

In order to further investigate the genomic‐wide profile of USP38, KDM5B, and H2Bub during LPS stimulation, we performed ChIP‐sequencing of USP38, KDM5B, and H2Bub by LPS stimulation. Consistently, USP38, KDM5B, and H2Bub co‐occupancy profiles were similar at *Il6*, and *Ltb* loci, but not at *Tnf* loci (Figure S6A, Supporting Information). Interestingly, the binding of USP38 to *Il23a* loci showed one peak at the start site on exon, while KDM5B and H2Bub showed multiple peaks along *Il23a* loci, besides the start site on exon (Figure S6A, Supporting Information), indicating that KDM5B and H2Bub might have additional regulatory function of *Il23a* transcription, besides their coregulation with USP38 during the LPS treatment. We then investigated putative recruitment of USP38, KMD5B, and H2Bub using DREME (discriminative regular expression motif elicitation) to discover consensus motif enriched over ChIP‐seq peaks, which revealed binding sequences predicted for LPS treatment. The most enriched motif of USP38 and KDM5B by GO terms analysis were inflammatory response and immune response, however, the most enriched motif of H2Bub were gene transcription (Figure S6B, Supporting Information). Moreover, by investigating chromatin association, USP38 and KDM5B mapping is correlated with H2Bub, with similar peak distribution (Figure S6C, Supporting Information). 21% of USP38‐associated genes were cotargeted by KDM5B (Figure S6D, Supporting Information), and USP38 and H2Bub cotargeted genes were enriched for gene ontology terms involved in gene transcription and damage signaling response (Figure S6E, Supporting Information). Overall, these data demonstrate the interplay of USP38 and KDM5B to regulate pro‐inflammatory cytokine gene expression through histone modifications.

### 2.8 USP38 Stabilizes KDM5B

**Figure 7:**
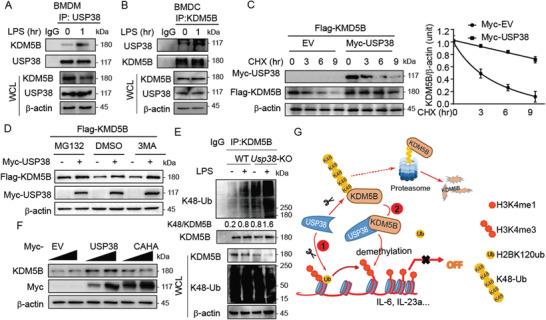
USP38 stabilizes KDM5B. A,B) Coimmunoprecipitation and immunoblot analysis of extracts of BMDMs and BMDCs with the indicated antibodies under LPS treatment for the indicated time points. C) Immunoblot analysis of extracts of 293T cells in the presence of USP38 along with Flag‐KDM5B (25 or 100 ng) and treated with cycloheximide (CHX) for the indicated time periods. The protein levels of KDM5B/*β*‐actin were quantified by ImageJ software (NIH). D) Immunoblot analysis of extracts of 293T cells transfected with Flag‐KDM5B along with or without Myc‐USP38, followed by treatment with DMSO, MG132, or 3‐methyladenine (3‐MA). E) Coimmunoprecipitation and immunoblot analysis of extracts of wild‐type (WT) or *Usp38*‐knockout (KO) BMDMs with or without LPS treatment. The protein levels of K48‐Ub/KDM5B were quantified by ImageJ software (NIH). F) Immunoblot analysis of extracts of 293T cells transfected with increasing doses of Myc‐empty vector (EV), Myc‐USP38, or Myc‐USP38‐CAHA mutant. G) Schematic representation of the working model of USP38 coupling with KDM5B to mediate the crosstalk between H2B monoubiquitination and H3 methylation in the regulation of inflammation. Data in (A)–(F) are representative of three independent biological experiments.

To further investigate the regulatory mechanism of KDM5B by USP38, we detected the interaction between USP38 and KDM5B and found that their interaction was enhanced by LPS stimulation in both BMDCs and BMDMs (**Figure** **7**A,B). In addition, we observed the decelerated degradation rate of KDM5B in the presence of USP38 by a cycloheximide‐chase (CHX) assay (Figure 7C). We next found that although the proteasome inhibitor MG132 does not completely diminish the effect of USP38 on the protein level of KDM5B, it largely reduced the difference of KDM5B protein abundance in the presence of USP38 or not (Figure 7D). Therefore, USP38 played a role to control KDM5B stability through proteasome pathway, although it might also affect KDM5B level by other mechanisms, which need further study in future. Since K48‐linked ubiquitin chains mainly serve as critical signals for proteasomal degradation, we examined whether USP38 stabilizes KDM5B by inhibiting its K48‐linked ubiquitination. We observed a marked accumulation of K48‐linked ubiquitination of KDM5B as well as a lower amount of KDM5B protein in *Usp38*‐KO BMDMs stimulated with LPS compared to WT BMDMs (Figure 7E). Furthermore, the USP38 inactive mutant no longer stabilized KDM5B (Figure 7F). Collectively, these data suggest that USP38 could stabilize histone demethylase KDM5B by cleaving its K48‐linked ubiquitin chains, thereby promoting the recruitment of KDM5B to chromatin during inflammation.

## 3 Discussion

Epigenetic regulation plays a fundamental role in the regulation of chromatin structure and gene expression. In particular, chromatin modifications, which are responsible for chromatin remodeling and specific gene transcription, are strictly controlled by different types of epigenetic modifiers through the posttranslational modifications of histones, including acetylation, methylation and ubiquitination.^[^ ^12^ ^]^ Histone methylation, ubiquitination and acetylation are reported to be involved in transcription by regulating the opening and closing of chromatin. There are many reports showing that acetylation of H3 at K9/14/18, ubiquitination of H2B at K120 or methylation of H3 at K4/36/79 is considered an activating signal for transcription, whereas methylation of H3 at K9/27 or ubiquitination of H2A at K119 is considered a repressing signal.^[^ ^27^ ^]^ In recent years, the functions of epigenetic modifiers in innate immune responses and related diseases have begun to emerge.^[^ ^28^ ^]^ A number of chromatin modifiers that control acetylation and methylation are critically involved in the transcriptional and posttranscriptional regulation of innate immune signaling pathways at multiple levels. For instance, methyltransferase SETD2 enhances antiviral immunity during infection by monomethylation of STAT1,^[^ ^50^ ^]^ and histone deacetylase HDAC9 can specifically deacetylate TBK1 to promote the antiviral response.^[^ ^51^ ^]^ Recently, histone modifications have also been reported to be involved in the regulation of the inflammatory response.^[^ ^42^, ^52^ ^]^ Histone deacetylase HDAC1/2 inhibits *Il6* transcription during the LPS response,^[^ ^14^ ^]^ while demethylase KDM6A/B decreases H3K27me3 to impair proinflammatory cytokine secretion.^[^ ^13^ ^]^ However, the function of histone ubiquitination in the regulation of the inflammatory response is poorly understood.

Despite its function of maintaining chromatin stability,^[^ ^53^ ^]^ histone ubiquitination is also classified as one of the prerequisites for histone methylation, which then triggers chromatin remodeling^[^ ^29^ ^]^ and gene expression,^[^ ^32^ ^]^ although the regulatory mechanism for this process is unclear. In past decades, several deubiquitinases such as USP3, USP7, USP15, USP22, USP44, and USP49 have been discovered to specifically remove the monoubiquitin of H2B.^[^ ^54^, ^55^, ^56^, ^57^, ^58^, ^59^ ^]^ However, little is known about the function of histone deubiquitinases in innate immunity and inflammation. Here, we identify a novel histone deubiquitination‐mediated repression of the inflammatory response by USP38. USP38 specifically cleaves the H2Bub, which enhances the demethylation of H3K4me3 on *Il6* and *Il23a* promoters, thus inhibiting their transcription, but have no effect on *Tnf* promoter. We reasoned that different categories of the pro‐inflammatory cytokine gene loci should have different histone modification patterns to meet their regulatory requirements. It has been showed that HDAC2 associates with Tet2 to specifically repress IL‐6, but not TNF*α* through deacetylation of H3Ac and H4Ac at *Il6* promoter in BMDMs.^[^ ^14^ ^]^ In addition, H3K27me3 demethylase, KDM6A and KDM6B, promoted IL6 but not TNF*α* expression through removal of H3K27me3 of its promoter.^[^ ^60^, ^61^ ^]^ Therefore, the modification of different gene locus could result in different transcript categories. As many pro‐inflammatory cytokines promoters are enriched with H3K4me3, the inhibition of H3K4me3 demethylation mediated by USP38 deficiency will enhance the inflammatory response. Consistent with our data at cell level, *Usp38‐*KO mice showed severe tissue damage and inflammatory cell infiltration in the DSS model, which confirms the regulatory function of H2Bub in the inflammatory response in vivo. Our findings reveal novel crosstalk between histone ubiquitination and methylation in the regulation of selective gene transcription as well as inflammatory responses via the USP38/KDM5B axis.

KDM5B is a lysine‐specific demethylase that specifically removes methylation from H3K4.^[^ ^62^ ^]^ KDM5B is involved in gene transcriptional repression.^[^ ^62^ ^]^ It also functions as an important player in DNA repair and genome stability in cancer cells.^[^ ^63^ ^]^ However, how KDM5B functions in the inflammatory response remains elusive. Here, we identify the role of KDM5B in repressing the inflammatory response by specifically reducing H3K4me3 in an H2Bub‐dependent manner with the help of USP38. Furthermore, we unexpectedly found that USP38 could prevent KDM5B from proteasomal degradation by cleaving its K48‐linked poly‐ubiquitin chains. Thus, USP38 has dual roles to facilitate the function of KDM5B in the regulation of histone demethylation and gene transcription: USP38 removes the inhibitory signal for KDM5B recruitment by cleaving H2Bub; on the other hand, USP38 stabilizes KDM5B and forms a complex with KDM5B on the promoters of *Il6* and *Il23a*. Thus, USP38 and KDM5B limit excessive inflammatory responses together by selective inhibition of proinflammatory cytokines, while loss of either *Usp38* or *Kdm5b* could enhance inflammatory responses (Figure 7G).

Taken together, our work illustrates the complex and precise control of inflammation by the cooperation between histone ubiquitination and methylation. Since loss of H2Bub has been reported in the pathogenesis of multiple cancers, including breast, colorectal, lung, and parathyroid cancers,^[^ ^53^ ^]^ the deubiquitinase USP38 might be a prominent epigenetic therapeutic target for inflammation, autoimmunity, and cancer in the future.

## 4 Experimental Section

##### Mice and Reagents

*Usp38*‐KO mice were generated by TALEN technology (Cyagen).^[^ ^64^ ^]^ All mice were breeding in a specific‐pathogen free (SPF) animal facility at Sun Yat‐sen University. All experiments were developed according to The Institutional Animal Care and Use Committee. The investigator performed the mice experiments was approved by Sun Yat‐sen University Laboratory Animal Center (2014‐22‐45).

##### Isolation and Transfection of Primary Cells

The isolation of BMDCs, BMDMs, and PMs was performed as described previously.^[^ ^64^ ^]^ Peripheral blood mononuclear cells (PBMCs) were isolated from blood from healthy donors (Zhongshan School of Medicine) as described.^[^ ^65^ ^]^ The use of PBMCs was in compliance with institutional guidelines and approved protocols by Sun Yat‐sen University, and all healthy donors signed a consent form approved by the Research Ethics Committee of the Sun Yat‐sen University Cancer Center (GZR2013‐040). The transfection of plasmid in *Usp38*‐KO BMDMs was done by jetPRIME (Polyplus‐transfection) according to the manufacturer's protocols.

##### Induction of Endotoxin Shock and DSS Colitis in Mice

To induce endotoxin shock, mice were intraperitoneally injected with LPS (10 mg kg^−1^ body weight, Sigma) and euthanized after 48 h. Mice injected with PBS vehicle were used as controls. To induce acute colitis, mice were administered 2.5% DSS (mol. wt. 35 000 to 50 000 Da; MP Biomedicals) in their drinking water, which was provided ad libitum, for 7 d and euthanized on day 8. In these mice, regular drinking water was used as the vehicle control. By neutralizing IL‐6/IL‐23, anti‐IL23 (anti‐mouse IL‐12/23 (p40) mAb C17.8, for neutralization, 3451‐5N‐500, MABTECH) (1 mg kg^−1^), and antimouse IL‐6 neutralizing antibody (mabg‐mil6‐3, InvivoGen) (1 mg kg^−1^) were injected intraperitoneally.

##### Bone‐Marrow Transplant

To perform bone marrow transplant, recipient mice (eight weeks old) were given antibiotic (Gentamicin, 1 mg mL^−1^) in drinking water for 2 d before given 1000r irradiation. Donor cells were obtained from long bones of donor mice (6–8 weeks old). ≈5 × 10^6^ cells were intravenous injected into the recipient mice (after irradiation) and followed with antibiotic drinking water for one week.

##### Clinical Scoring and Histological Assessment of the Mouse Model

In the endotoxin shock mouse model, mice were injected with LPS, blood was assessed at 18 h, and mice were euthanized at 48 h to remove the lung for H&E staining as described.^[^ ^14^ ^]^ In the colitis model, during the course of experiments, body weight and the presence of occult blood were determined daily beginning on day 3. Mice were scored for stool consistency (0–3), presence of blood in stool (0–3), and general appearance (0–3) as described previously.^[^ ^37^ ^]^ These scores were added to generate a total clinical score ranging from 0 to 9. After euthanasia, the entire colon was quickly removed from each mouse, and the length of each colon was measured. The removed colons were fixed in 4% paraformaldehyde for 24 h, embedded in paraffin, and then stained with H&E as previously described.^[^ ^66^ ^]^ Histology was scored by an investigator in a blinded fashion as a combination of inflammatory cell infiltration (score 0–3) and tissue damage (score 0–3) with a total score ranging from 0 to 6.

##### Colon Organ Culture

Colons from mice were assessed and treated as previously described.^[^ ^66^ ^]^ Supernatants were collected for cytokine detection.

##### ChIP and Re‐ChIP Assay

ChIP assays in BMDCs or BMDMs (1 × 10^8^) were stimulated with LPS for the indicated time points and then fixed for the experiments according to the protocol described in SimpleChIP Enzymatic Chromatin IP Kit (CST, No.9003). Re‐ChIP assays were performed by two times IP with different antibody as described previously.^[^ ^67^ ^]^ ChIP assays were performed with BMDMs (1 × 10^7^) with or without LPS stimulation for 4 h. 1% input DNA and ChIP‐IgG (rabbit) were assessed for comparison and quality control. For CHIP‐seq analysis, raw reads were mapped to the mouse reference genome (mm10) using bowtie2. The aligned SAM files were filtered to remove PCR duplicate reads and converted to sorted BAM files using SAMtools. Peak calling was performed with MACS2 using a false discovery rate threshold set to ≤0.05. Peaks were annotated to the closest transcription start sites (TSS) and gene features using the ChIPSeeker R package. Motif analysis was performed by DREME. Functional enrichment in GO biological processes of target genes was performed using clusterProfiler R package.

##### Fractionation of Bound and Unbound Chromatin

Cells were resuspended in buffer A (5 × 10^−3^ m HEPES pH 7.9, 0.75 × 10^−3^ m MgCl_2_, 5 × 10^−3^ m KCl, 0.25 × 10^−3^ m dithiothreitol [DTT]), and incubated on ice for 30 min and lysed with Dounce homogenizer. Cells were then centrifuged for 15 min at 3300 *g*/4 °C, transfer the supernatant (S1) to a new tube and resuspend the pellet with buffer B (buffer A supplemented with 0.34 m sucrose, 10% glycerol, 10 × 10^−3^ m NaF, and 1 × 10^−3^ m Na_3_VO_4_) plus 0.5% NP40 and incubated on ice for 30 min followed with 10 min centrifuge at 3300 *g*/4 °C. Supernatant was add to (S1) as the “unbound” fraction and the pellet was the “bound” fraction.^[^ ^58^ ^]^

##### In Vitro Deubiquitination Assay

Cells overexpressed with Flag‐H2B were incubated with buffer A (0.25 m sucrose, 60 × 10^−3^ m KCl, 15 × 10^−3^ m NaCl, 10 × 10^−3^ m MES, pH 6.5, 5 × 10^−3^ m MgCl_2_, 1 × 10^−3^ m CaCl_2_, 0.5% Triton X‐100, 1 × 10^−3^ m DTT, 0.1 × 10^−3^ m PMSF) to release nuclei and then extract nucleosomes from nuclei with RIPA, subjected these nucleosomes to immunoprecipitation with anti‐Flag beads and eluted with Flag peptide. Incubated nucleosomes with Myc‐USP38 or Myc‐USP38 CAHA derived from 293T cells in deubiquitination reaction buffer B (100 × 10^−3^ m Tris‐HCl, pH 8.0, 1 × 10^−3^ m EDTA, 0.1 × 10^−3^ m PMSF, 1 × 10^−3^ m DTT plus Protease Inhibitor) at 37 °C for 45 min. Reaction was terminated by addition of loading buffer and detected by immunoblotting.^[^ ^68^ ^]^

##### ChIP‐qPCR Analysis

Each immunoprecipitation was repeated from at least three different extracts. Immunoprecipitated DNA was analyzed by quantitative PCR. Primers for ChIP‐qPCR were designed using Snapgene software and in silico validated in UCSC genome browser for specificity. All signals were normalized and calculated using 1% input and used for comparison between experimental samples.^[^ ^14^ ^]^ Calculation of the percent of input for each ChIP was shown: %Input = 2^(−ΔCt [normalized ChIP])^. The primer pairs were as follows:

IL‐6: (5′CCTGCGTTTAAATAACATCAGCTTTAGCTT3′, 5′GCACAATGTGACGTCGTTTAGCATCGAA3′)
TNF‐*α*: (5′CCAGCCAGCAGAAGCTCCCTCAGCGAG3′, 5′GCGGATCATGCTTTCTGTGCTCATGGTGTC3′)
IL‐23*α* p19: (5′CCTCTAGCCACAACAACCTC3′, 5′CCTTCACACTAGCAGGTGACT3′)

##### RNA‐seq Analysis

Total RNA was extracted by Trizol (Invitrogen, 10296010), and the RNA sequencing was performed using the Illumina platform by Novogene Company. Quality control of the fastq data was performed with FASTQC. High quality reads were aligned to the mouse reference genome (mm10) using histat2.^[^ ^69^ ^]^ The aligned SAM file was converted into sorted BAM files by SAMtools, and the HTSeq‐count program was used to count the total number of reads that are mapped to the genome. The differential gene expression analysis was performed by DESeq2 R package. Gene enrichment was analyzed by clusterProfiler R package.

##### Statistical Analysis

The results of all quantitative experiments are reported as mean ± SEM of three independent experiments, data are reported as means ± SD of six mice per group, Mantel–Cox test and Gehan–Breslow–Wilcoxon test were used for mice survival analysis, and Student's *t*‐test was used for all statistical analyses with the GraphPad Prism 8.0 software.

## Conflict of Interest

The authors declare no conflict of interest.

## Author Contributions

Z.Z., Z.S., and D.L. performed the experiments and analyzed the results. P.L., Y.S., F.J, W.Y., M.L., W.C., Z.X., and J.H. provided technical help. J.C. initiated and designed the project and directed the research. Z.Z. and J.C. wrote the manuscript.

## Supporting information

Supporting Information
