# Supplementary material for: USP38 Couples Histone Ubiquitination and Methylation via KDM5B to Resolve Inflammation
Source: Adv Sci (Weinh). 2020 Oct 11;7(22):2002680. doi: 10.1002/advs.202002680 (PMC7675183; doi:10.1002/advs.202002680)
Supplement: Supplementary file 1 — Supporting Information [file ADVS-7-2002680-s001.pdf]

WILEY-VCH

© 2020 Wiley-VCH GmbH

ADVANCED SCIENCE
Open Access

Supporting Information

for *Adv. Sci.*, DOI: 10.1002/advs.202002680

USP38 couples histone ubiquitination and methylation via KDM5B to resolve inflammation

Zhiyao Zhao[1,2], Zexiong Su[1], Puping Liang[1], Di Liu[1], Shuai Yang[1], Yaoxing Wu[1], Ling Ma[1], Junyan Feng[1], Xiya Zhang[1], Chenglei Wu[1], Junjiu Huang[1], Jun Cui[1,*]

Supporting Information

USP38 couples histone ubiquitination and methylation via KDM5B to resolve inflammation

Zhiyao Zhao[1,2], Zexiong Su[1], Puping Liang[1], Di Liu[1], Shuai Yang[1], Yaoxing Wu[1], Ling Ma[1], Junyan Feng[1], Xiya Zhang[1], Chenglei Wu[1], Junjiu Huang[1] and Jun Cui[1]*

[1] MOE Key Laboratory of Gene Function and Regulation, State Key Laboratory of Biocontrol, School of Life Sciences, Sun Yat-sen University, Guangzhou, Guangdong, 510006, China

[2] Department of Internal Medicine, Guangzhou Institute of Pediatrics, Guangzhou Women and Children's Medical Center. Guangzhou, Guangdong, 510623, China

*Correspondence should be addressed to J.C. (cuij5@mail.sysu.edu.cn)

Contents of supporting information

Supplementary Figure 1-6

Extended experimental procedures

Figure S1

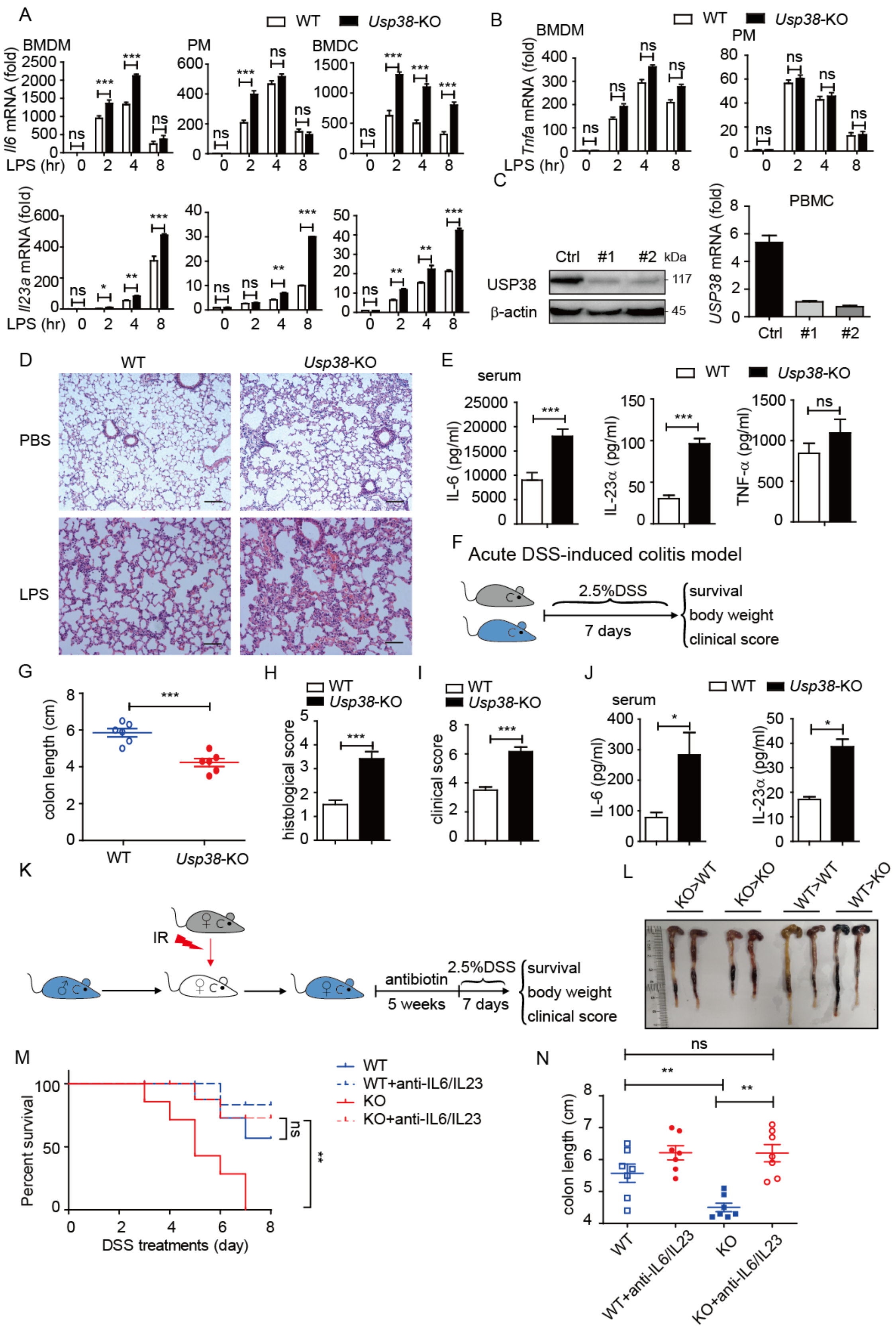
A
WT
Usp38-KO
BMDM
PM
BMDC
Il6 mRNA (fold)
Il23a mRNA (fold)
LPS (hr)
B
Tnfa mRNA (fold)
C
PBMC
Ctrl
#1
#2
kDa
USP38
β-actin
117
45
USP38 mRNA (fold)
D
PBS
LPS
E
serum
IL-6 (pg/ml)
IL-23α (pg/ml)
TNF-α (pg/ml)
F
Acute DSS-induced colitis model
2.5%DSS
7 days
survival
body weight
clinical score
G
colon length (cm)
H
histological score
I
clinical score
J
K
IR
antibiotin
5 weeks
L
KO>WT
KO>KO
WT>WT
WT>KO
M
WT+anti-IL6/IL23
KO
KO+anti-IL6/IL23
Percent survival
DSS treatments (day)
N

Figure S1, Loss of USP38 maintains a higher inflammatory response. (A) Expression levels of *Il6* and *Il23a* mRNA in BMDMs and PMs from WT and *Usp38*-KO mice during LPS stimulation for the indicated time points. **(B)** Expression level of *Tnfa* in BMDMs and PMs from WT and *Usp38*-KO mice during LPS stimulation for the indicated time points. **(C)** Knockdown efficiency of *USP38* by *USP38*-specific siRNAs was evaluated by qPCR and immunoblotting in PBMCs. **(D)** Histopathology of lungs from WT or *Usp38*-KO mice after intraperitoneal injection of LPS (10 mg/kg body weight) for 18 hr. Scale bars, 20 μm. **(E)** Protein levels of IL-6 and TNF-α in serum from WT or *Usp38*-KO mice after intraperitoneal injection with LPS (10 mg/kg body weight) for 4 hr. **(F)** Schematic illustration of the dextran sulfate sodium (DSS)-induced acute colitis model. **(G-I)** Colon length **(G)**, histological score **(H)** and clinical score **(I)** of WT and *Usp38*-KO mice of acute colitis. **(J)** Protein levels of IL-6 and IL-1β in serum from **(G)**. **(K)** Schematic illustration of the construction of bone marrow chimera mice. **(L)** The colon length present in DSS-induced colitis from chimera mice. **(M-N)** Survival **(M)** and colon length changes **(N)** in WT and *Usp38*-KO mice followed with anti-IL6/IL-23 Abs (1mg/kg) treatment. n=7.

Data in **A-C** are presented as the means ± SEM of at least three biological experiments. Data in **E, G-J, N** are presented as the means ± SD of the experiment with six/seven mice per group. Data in **D** and **L** are representative of 3 independent biological experiments. *p < 0.05, **p < 0.01, ***p < 0.001, ns, no significant difference, versus the wild-type or control group with the same treatment (Student's *t*-test in **A**-**C**, **E**, **H**-**J**, **N** and Mantel-Cox test and Gehan-Breslow-Wilcoxon test in **M**).

Figure S2

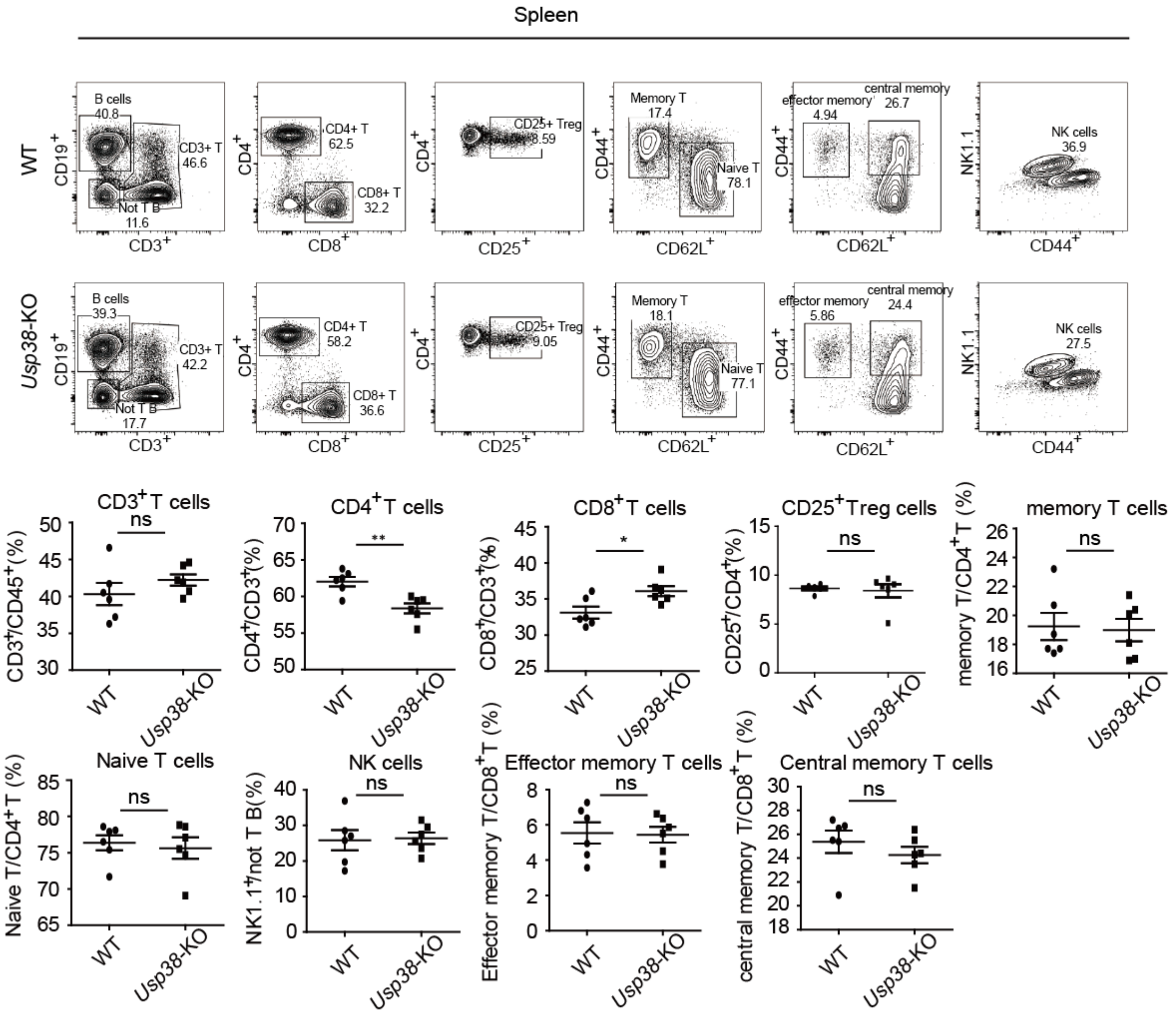

Figure S2, Loss of USP38 has limited effects on the development of T cells. The percentages of the indicated T cells were analyzed by their specific lineage markers from WT and *Usp38*-KO mice. Data are presented as the means ± SD of the experiment with six mice per group. *p < 0.05, **p < 0.01, ns, no significant difference, versus the WT group (Student's *t*-test).

Figure S3

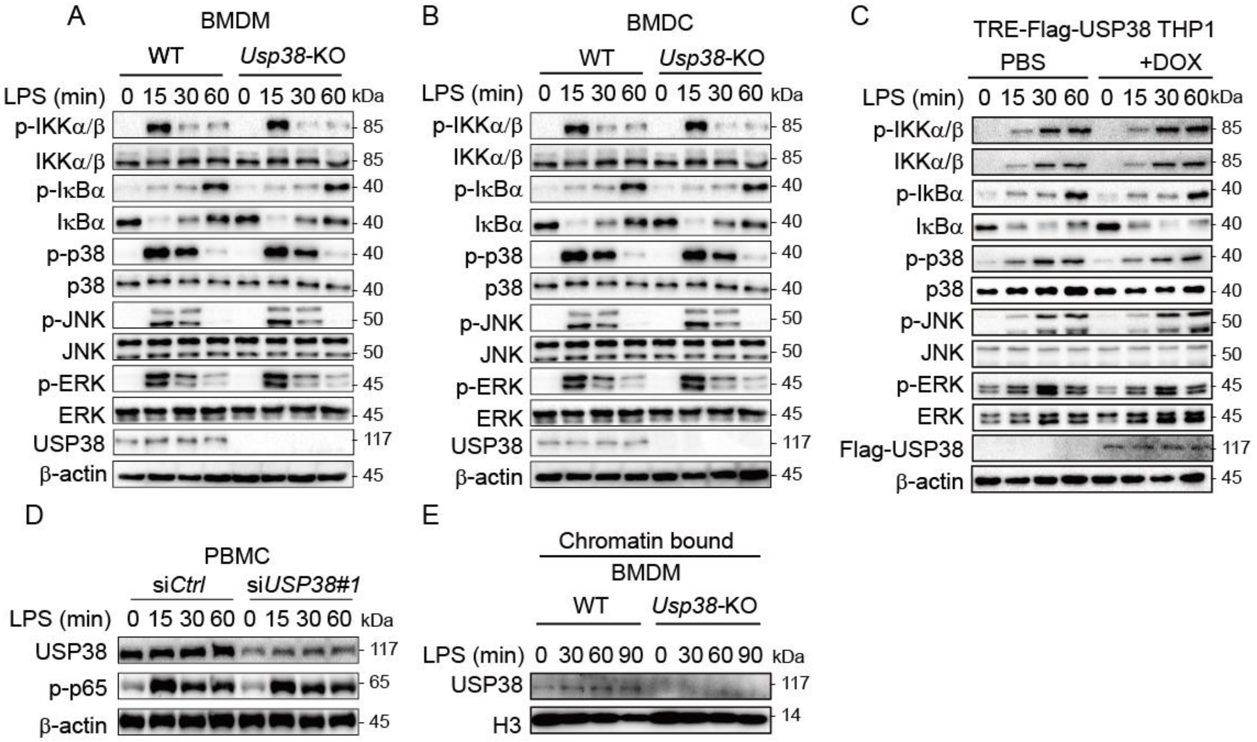

Figure S3, USP38 is dispensable for the TLR-stimulated signaling cascade. (A-B) Immunoblot analysis of phosphorylated (p-) and total proteins in whole-cell lysates of WT and *Usp38*-KO BMDMs **(A)** and BMDCs **(B)** that were stimulated with LPS for the indicated time points. **(C)** Immunoblot analysis of phosphorylated (p-) and total protein in whole-cell lysates of USP38-inducible THP1 cells with or without DOX that were stimulated with LPS for the indicated time points. **(D)** Immunoblot analysis of phosphorylated p65 in PBMCs silenced with control siRNA (si*Ctrl*) or *USP38*-specific siRNA (si*USP38*) under LPS treatment for the indicated time points. **(E)** Immunoblot analysis of USP38 in the chromatin-bound and unbound components of WT and *Usp38*-KO BMDMs under LPS treatment for the indicated time points. Data are representative of 3 independent biological experiments.

Figure S4

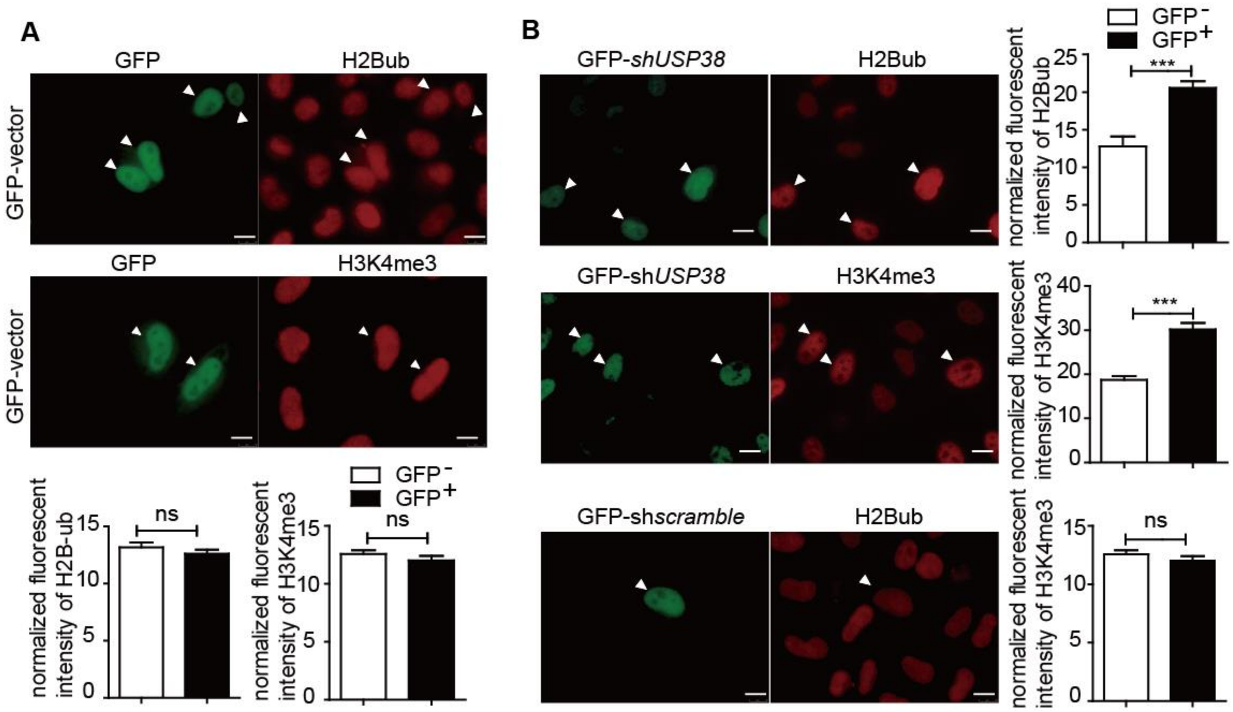

Figure S4, USP38 specifically reduces the mono-ubiquitination of H2B and tri-methylation of H3K4. (A) Immunofluorescence analysis of H2Bub and H3K4me3 with GFP-vector in HeLa cells. The same experiments were performed three times. The relative intensity of fluorescence signals of H2Bub and H3K4me3 in GFP vector-transfected cells (GFP^+) vs. control cells (GFP^-) in the same image frame and were analyzed by ImageJ software (NIH). **(B)** Immunofluorescence analysis of H2BK120ub and H3K4me3 with *USP38*-silenced *USP38*-specific shRNA or with sh*scramble* RNA (with GFP as a marker in the same plasmid) in HeLa cells. The same experiments were performed three times. The relative intensity of fluorescence signals of H2Bub and H3K4me3 in shRNA-transfected cells (GFP^+) vs. control cells (GFP^-) in the same image frame and were analyzed by ImageJ software (NIH). Data in **A-B** are presented as the means ± SEM of at least three independent biological experiments with 50 cells/experiment. ***$p < 0.001$, ns, no significant difference, versus the control cells (Student's *t*-test).

Figure S5

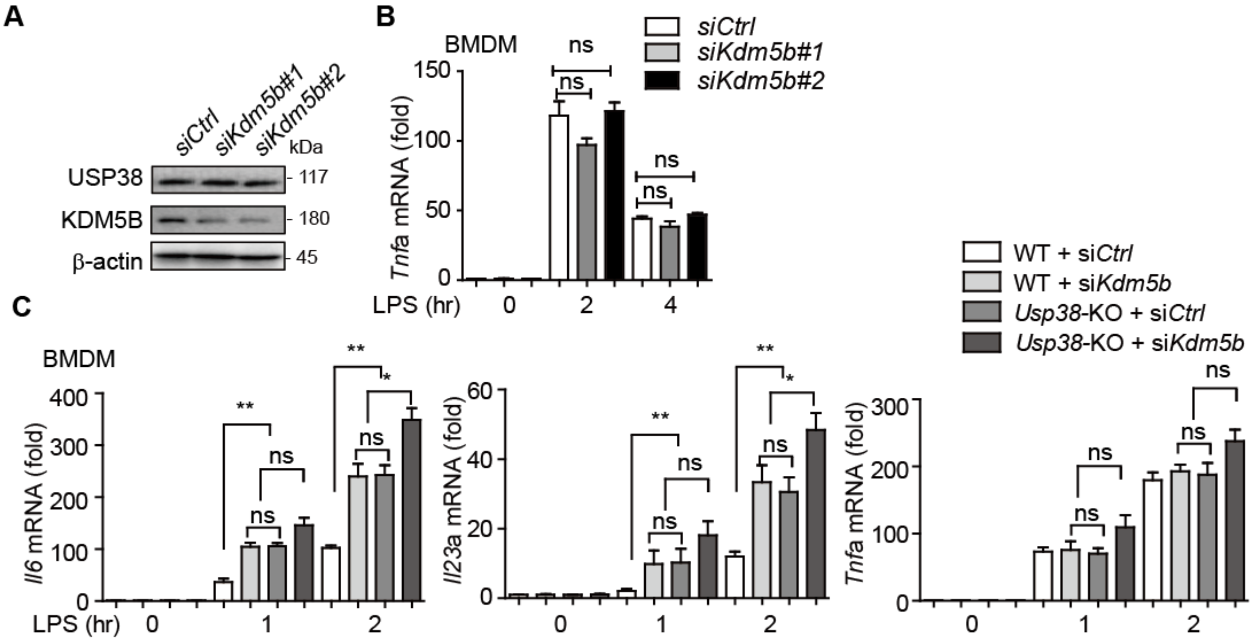

Figure S5, USP38 recruits KDM5B to specifically repress the transcription of *Il6* and *Il23a*.

(A) Immunoblot analysis of KDM5B and USP38 in *Kdm5b*-silenced BMDMs with *Kdm5b*-specific siRNAs (*siKdm5b#1*, *siKdm5b#2*) or control siRNA (*siCtrl*). **(B)** The level of *Tnfa* mRNA in WT BMDMs transfected with or without *Kdm5b*-specific siRNAs under LPS treatment for the indicated time points. **(C)** The levels of *Il6*, *Il23a* and *Tnfa* mRNA in WT or *Usp38*-KO BMDMs with *Kdm5b*-specific siRNA or control siRNA under LPS stimulation for the indicated time points. Data in **A** are representative of 3 independent biological experiments. Data in **B-C** are presented as the means ± SEM of at least three independent biological experiments. *p < 0.05, **p < 0.01, ***p < 0.001, ns, no significant difference, versus the indicated group with the same treatment (Student's *t*-test).

Figure S6

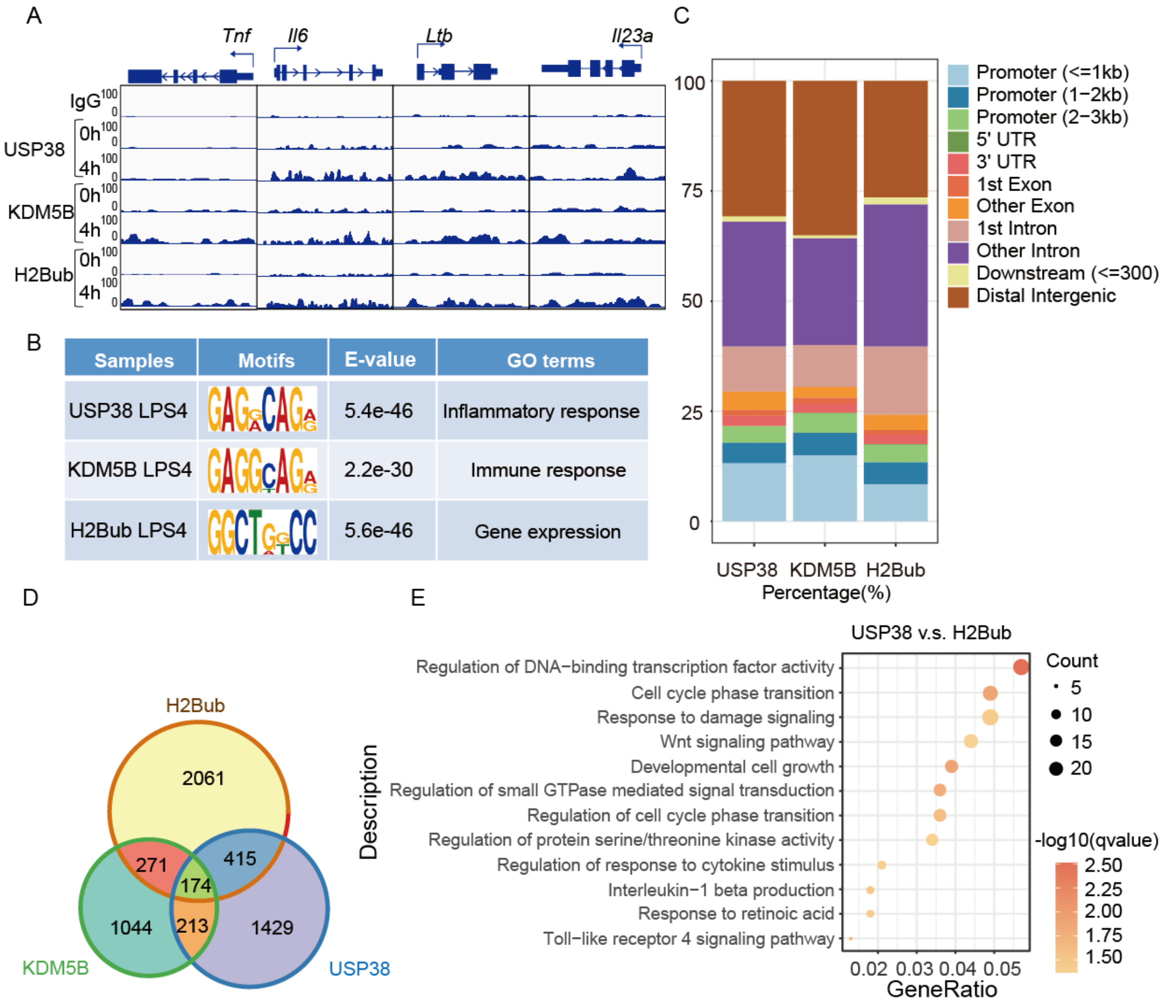

Figure S6, USP38 and KDM5B chromatin association and H2Bub enrichment during LPS stimulation. (A) IGV browser tracks show the distribution of USP38, KDM5B and H2Bub ChIP-seq signals at indicated loci in BMDMs with or without LPS stimulation for 4 hours. IgG was added for quality control. **(B)** Sequence motif identified within top enriched genes regulated by USP38, KDM5B and H2Bub, followed with GO terms analysis. **(C)** Genome-wide compartment analysis of ChIP–seq of USP38, KDM5B and H2Bub in genome-wide peak distribution. **(D)** Overlap of enriched genes targeted by USP38, KDM5B and H2Bub in BMDMs. **(E)** Gene Ontology enrichment analysis performed on common USP38–H2Bub targets.

EXTENDED EXPERIMENTAL PROCEDURES

Cell culture

HEK 293T, THP-1 cells, human peripheral blood mononuclear cells (PBMCs), bone marrow derived macrophages (BMDMs), and bone-marrow derived dendritic cells (BMDCs) were cultivated in Dulbecco's modified Eagle's medium (HyClone) or RPMI-1640 medium (Gibco) supplemented with 10% fetal bovine serum (GenStar) and 1% L-glutamine (Gibco) at 37°C in 5% CO_2.

Reagents

Expression plasmids were transfected with StarFect High-efficiency Transfection Reagent (GenStar) according to the manufacturer's instruction. Puromycin (P9620), MG132 (C-2211-5MG) (10μM), bafilomycin A1 (H2714) (0.2mM), and 3-methyladenine (3-MA) (M9281-100MG) (10μM), lipopolysaccharides (LPS, L4391-1MG), cycloheximide from microbial (CHX, C1988-1g) were purchased from Sigma.

Antibodies

Anti-c-Myc-HRP (11814150001) were purchased from Roche Applied Science. Anti-H2B (12364S), anti-H2A (12349S), K48-linkage Specific Polyubiquitin antibody (4289) were purchased from CST. Anti-H3K4me3 (A2357) was purchased from Abclonal. Anti-H3K9me3 (61013) was purchased from Active motif. Anti-H3K4me3 (A2357) was purchased from

Abclonal. Anti-Histone H3 antibody (ab1791) and anti-KDM5B (ab181089) were purchased from Abcam. Anti-USP38 antibody (17767-I-AP) was purchased from Proteintech. Anti-β-Actin (A2228) was purchased from Sigma. CD44-FITC (11-0445-82), CD3-PE (12-0038-42), CD19-FITC (11-0193-82), CD8a-PerCP-Cyanine5.5 (45-0081-82), CD4-APC (17-0041-82), CD25-PerCP-Cyanine5.5 (45-0251-82), CD62L-PE-Cyanine7 (25-0621-82) and NK1.1- PerCP-Cyanine5.5 (45-5941-82) were purchased from eBioscience.

RNA Extraction and Quantitative RT-PCR

Total RNA was isolated by TRIzol Reagent (Invitrogen). HiScript III RT SuperMix for qPCR (R323-01) was purchased from Vazyme. SYBR Green qPCR Mix (GenStar) was applied for Real-time PCR, and data were normalized to GAPDH. Primers used for real-time PCR were followed:

mIl6 forward primer: 5’ TAGTCCTTCCTACCCCAATTTCC 3’

mIl6 reverse primer: 5’ TTGGTCCTTAGCCACTCCTTC 3’

mTnfa forward primer: 5’ CCCTCACACTCAGATCATCTTCT 3’

mTnfa reverse primer: 5’ GCTACGACGTGGGCTACAG 3’

mIl23a forward primer: 5’GCCAAGAAGACCATTCCCGA3’

mIl23a reverse primer: 5’TCAGTGCTACAATCTTCTTCAGAGGACA3’

mIrf4 forward primer: 5’CCGACAGTGGTTGATCGACC3’

mIrf4 reverse primer: 5’CCTCACGATTGTAGTCCTGCTT3’

mGAPDH forward primer: 5'GAAGGGCTCATGACCACAGT 3'

mGAPDH reverse primer:5' GGATGCAGGGATGATGTTCT 3'

Measurement of cytokines

Concentrations of the cytokines in cell culture supernatants or mouse serum were determined by ELISA kits (BD Biosciences), according to the manufacturer's recommendations.

Immunoprecipitation and Immunoblotting

For immunoprecipitation, cells were collected and digested by low salt lysis buffer (LSB, 50 mM HEPES pH 7.5, 150 mM NaCl, 1 mM EDTA, 1.5 mM MgCl2, 10% glycerol, 1% Triton X-100) plus protease inhibitors on ice for 30 minutes. Cell lysates were incubated overnight with anti-Flag or anti-Myc agarose gels (Sigma) or the appropriate antibodies plus Protein A/G beads (Pierce). Then beads were washed for 5 times with LSB and eluted with 2×SEMS Loading Buffer (Cell Signaling Technology) and detected by immune-blotting and incubated with corresponding antibodies for further analysis.

Immunofluorescence Staining

Cells were washed by PBS and fixed by 4% paraformaldehyde for 15 minutes and washed with cold PBS for two times before permeabilized with methyl alcohol for 10 min at -20°C. Cells were washed with cold PBS for three times and then blocked cells in 6% goat serum for 1 hour at

room temperature. Cells were incubated with primary antibody overnight, and incubated with fluorescently labeled secondary anti-body (Alexa Fluor 488- and Alexa Fluor 568-conjugated antibodies against mouse, rabbit or goat IgG (Biotium)) for 1 hour. Nuclei were stained with DAPI (Invitrogen). Pictures were captured by Ultrahigh Resolution Confocal Laser Microscope (Leica TCS SP8 STED 3X).
